# The structure of psychological life satisfaction: insights from farmers and a general community sample in Australia

**DOI:** 10.1186/1471-2458-12-976

**Published:** 2012-11-14

**Authors:** Léan V OBrien, Helen L Berry, Anthony Hogan

**Affiliations:** 1 National Centre for Epidemiology and Population Health, The Australian National University, Canberra, Australia; 2 Centre for Research and Action in Public Health, Faculty of Health, University of Canberra, Canberra, Australia; 3 School of Sociology, The Australian National University, Canberra, Australia

## Abstract

**Background:**

Psychological life satisfaction is a robust predictor of wellbeing. Public health measures to improve wellbeing would benefit from an understanding of how overall life satisfaction varies as a function of satisfaction with multiple life domains, an area that has been little explored. We examine a sample of drought-affected Australian farmers and a general community sample of Australians to investigate how domain satisfaction combines to form psychological satisfaction. In particular, we introduce a way of statistically testing for the presence of “supra-domains” of satisfaction to propose a novel way of examining the composition of psychological life satisfaction to gain insights for health promotion and policy.

**Methods:**

Covariance between different perceptions of life domain satisfaction was identified by conducting correlation, regression, and exploratory factor analyses on responses to the Personal Wellbeing Index. Structural equations modelling was then used to (a) validate satisfaction supra-domain constructs emerging from different perceptions of life domain satisfaction, and (b) model relationships between supra-domains and an explicit measure of psychological life satisfaction.

**Results:**

Perceived satisfaction with eight different life domains loaded onto a single unitary satisfaction construct adequately in each sample. However, in both samples, different domains better loaded onto two separate but correlated constructs (‘supra-domains’): “satisfaction with connectedness” and “satisfaction with efficacy”. Modelling reciprocal pathways between these supra-domains and an explicit measure of psychological life satisfaction revealed that efficacy mediated the link between connectedness and psychological satisfaction.

**Conclusions:**

If satisfaction with connectedness underlies satisfaction with efficacy (and thus psychological satisfaction), a novel insight for health policy emerges: psychological life satisfaction, a vital part of wellbeing, can potentially be enhanced by strengthening individuals’ connectedness to community. This may be particularly important and efficacious for vulnerable populations.

## Background

Psychological life satisfaction is an important and robust predictor of human health. However, little is understood about what life satisfaction comprises as a psychological concept. Studies have established that psychological life satisfaction is based on perceived satisfaction with a range of different life domains. Yet the mechanism by which these perceptions contribute to generalised psychological satisfaction has barely been examined. Consequently, it is difficult to affect psychological life satisfaction and this limits its practical utility when designing health promotion programs and preventative strategies. The current study examined and quantified the structure of psychological life satisfaction within two samples: farmers (a population at risk for mental health problems) and a general community sample of Australians. The results (i) reveal a simple architecture underpinning the way satisfaction on domains combines into psychological satisfaction and (ii) discuss how quantifying the structure of psychological life satisfaction provides practical insights for health promotion.

### Life satisfaction

Psychological life satisfaction is correlated with a range of health behaviours and outcomes, including smoking, harmful levels of drinking, anxiety, depression and suicide, and it can predict mental and physical health for up to twenty years [1-3]. Levels of psychological life satisfaction reflect people’s assessment of their life as it is, compared to life as they wish it were. It is related to (but distinct from) happiness, which refers to transitory levels of affect across time [4].

Despite the complexity of the concept, psychological life satisfaction is often measured using a single item e.g. [5]. However, there is a general consensus that psychological satisfaction is formed from perceptions of satisfaction with multiple life domains [6-8]. A heterogeneous range of domains has been examined and some researchers have sought to develop a comprehensive set [9]. Personal Wellbeing Index (PWI) is notable among these, because it has been iteratively developed over time [10] and, through the International Wellbeing network [11], it is now being used as the basis of an international effort to develop a standardised cross-cultural measure of life satisfaction. The PWI parsimoniously measures eight domains of satisfaction: achieving in life, future security, standard of living, health, safety, religion/spirituality, relationships and community connectedness [12-14]. Cummins and colleagues have shown these domains are inter-correlated, and correlated with overall psychological life satisfaction, while cross-cultural research has validated the scale in several countries including Algeria [15] and China [16].

### Population satisfaction: farmers versus the general community

Farmers are a population that could particularly benefit from a better understanding of the dynamics of life satisfaction. With the advent of climate change, countries including Australia, China and the United States of America will face periods of prolonged and intense dryness, placing farmer populations at risk for greater incidence of health problems [17]. Farmers are not simply financially vulnerable to the effects of climate variability, remoteness limits their access to health services, and this problem is compounded by a traditional reluctance to acknowledge the health problems; especially mental health problems [18,19]. However, farmers are willing to discuss level of psychological life satisfaction.

Unfortunately, existing research into domain and overall psychological life satisfaction for farmers versus the general community has yielded mixed results [20,21] and ways to address low satisfaction are still under theorised. Recently, researchers have begun to examine ways to improve life satisfaction, rather than purely using it as an effective sentinel measure of health and wellbeing. Thus far, research has examined the relationship between satisfaction and several different factors including participation in multiple social groups [22], being in a relationship [23], employment [23] and public welfare spending [24]. However, as it is currently understood, psychological life satisfaction is not very informative about how farmers’ distinct characteristics should be incorporated into health promotion programmes and policy.

The objective of our study was to identify key concepts and relationships that can be used to predict, affect and understand psychological life satisfaction in context, with a view to promoting life satisfaction, and thereby public health. As a population increasingly at risk from climate change, we examined Australian farmers and compared them to a general community sample of Australians using the PWI index. Our approach was to quantify the way that perceived satisfaction with different life domains combines into an overall sense of psychological life satisfaction.

### Quantifying the structure of life satisfaction

The process by which combined domain satisfaction forms overall psychological satisfaction is typically conceived in terms of simple addition [9]. However, a few studies have argued that some domains may have disproportionate influence on overall satisfaction. Mixed results have been found when asking individuals to explicitly weight the importance of different domains [25-27], but one study has demonstrated that the ratio by which different domains contribute to overall satisfaction can be determined statistically [9].

The novel approach we took to quantification was to consider how perceptions about satisfaction with life domains may conceptually overlap or co-vary in quantifiable ways *prior* to combining into psychological life satisfaction. Conceptual overlap in the perception of life domains is suggested by intersecting lines of research on social capital and personal efficacy. Social capital is a broad term capturing the concept of beneficial connection to others through social participation and social cohesion, and it is positively related to life satisfaction [28]. Three PWI domains: community connectedness, relationships and religious participation are all empirically established as components of social capital [29]. Personal efficacy refers to the perceived power to perform challenging tasks and achieve goals, and the PWI domains achieving in life and future security are elements of personal efficacy [30]. Consequently, we hypothesised that perceived domain satisfaction would combine into overarching ‘supra-domains’ that have distinct and separable relationships with psychological life satisfaction.

We did not make a firm prediction about the number of supra-domains that may occur since the role of perceived satisfaction with some PWI domains (standard of living, health and safety) was ambiguous. However we did expect that the eight discrete domains would combine into at least two supra-domains, “efficacy” and “connectedness”. Once modelled, such supra-domains can be tested for unique relationships with each other and with psychological life satisfaction. We therefore tested the hypotheses that:

1. *(i)* Satisfaction with domains will cohere into psychological life satisfaction. *(ii)* Some domains will be more influential than others.

2. Before cohering into psychological life satisfaction, domains will first combine into supra-domains, like “efficacy” and “connectedness”.

3. Supra-domains will have unique relationships with each other and with psychological satisfaction.

## Methods

### Study sample

The data were originally collected to inform the National Review of Drought Policy, undertaken by the Drought Review Branch of the Australian Government Department of Agriculture, Fisheries and Forestry [31]. In keeping with Australian Government regulations, the Australian Bureau of Statistics’ Statistical Clearing House acted as an external ethics committee, reviewed the survey for ethical concerns, and granted approval for its conduct. In June of 2008, under the direction of the Australian Bureau of Rural Sciences, the polling firm Newspoll collected data from two different samples: drought-affected farmers (N=500) and Australian adults (N=1,203; for more detail, see Appendix A in Additional file 1). Variables had 0–2 % missing data with the exception of household income and satisfaction with religion/spirituality (approximately 15-20% missing data in both samples). To avoid loss of power, missing data were imputed using Expectation Maximisation [32]; results for household income and satisfaction with religion/spirituality were treated with caution.

### Measurements

#### Socio-demographics

In addition to measures of life satisfaction, the survey included some socio-demographic measures: age (ordinal scale: 18–19 , 20–24, 25–29, 30–34, 35–39, 40–44, 45–49, 50–54, 55–59, 60–64 and 65+), sex (male/female), relationship status (married/not married), location (major city with population >1,000,000/remainder of State) employment (full-time/part-time/not in workforce), and household income (ordinal scale: under $30,000, $30,000-39,999, $40,000-49,999, $50,000-59,999, $60,000-69,999, $70,000-79,999, $80,000-89,999, $90,000-99,999, $100,000+). For analytical purposes, the ordinal scales were treated as interval data; household income was equivalised using the OECD method [33].

#### Personal well-being index

The PWI contains two parts: a single item measuring psychological life satisfaction (“How satisfied are you with your life as a whole”); and eight items, each measuring satisfaction with a different domain (“How satisfied are you with your…”: standard of living, health, achieving in life, relationships, safety, community connectedness, future security and religion/spirituality). Participants were asked to respond to each item using an 11-point scale (0=completely dissatisfied, 10=completely satisfied). Following Cummins and Nistico [6], responses were then converted ((x/11)*100) into scores ranging from 0–100, so that, for example, a score of “75” equates with “75% satisfied”.

### Statistical analysis

Descriptive statistics were generated for both samples and regressions adjusted for demographic characteristics compared cross-sample mean differences. Then, separately for farmer and general community samples, we undertook: (i) unadjusted correlations to establish bivariate relationships between satisfaction domains; (ii) multiple regression analyses to assess the level of unique versus shared variance in psychological satisfaction explained by different domains; (iii) exploratory factor analysis with maximum likelihood factor extraction and oblimin rotation to explore the presence of correlated supra-domains; and (iv) multiple regression analysis to replicate mediation pathways identified by structural equation modelling (SEM). Note that multi-group analyses of the SEM models indicated that the factor weightings and/or conceptual structure of life satisfaction was different across the two populations, making separate modelling the appropriate method of analysis.

SEM was performed using asymptotic distribution-free analysis (to allow for non-normally distributed data) and the following fit statistics: Chi-squared test, CFI score and RMSEA score. Note that RMSEA <=.05 was the core criterion used to assess models because it is less sensitive to sample size than the Chi-squared test and less sensitive to parameter number than CFI. SEM incorporates multiple techniques, including one-factor congeneric modelling (to confirm the factor structure of individual latent, or ‘unitary’, constructs), confirmatory factor analysis (to confirm relationships among latent constructs) and structural modelling (to show how multiple latent constructs may be causally related). We used these methods to test: (i) whether satisfaction was a unitary construct; (ii) whether supra-domains suggested by the exploratory factor analysis were unitary constructs; (iii) relationships between supra-domains; (iv) whether socio-economic variables could be helpfully summarised as latent constructs or should be used as separate variables; and then to (v) model multiple opposing pathways between supra-domains and overall psychological satisfaction, adjusting for socio-economic variables. In all modelling, non-significant items and pathways were deleted one-by-one and modification indices were used to add logically possible pathways until the final model reached best possible fit. Model fit was comprehensively re-evaluated after each deletion or modification and the Akaike Information Criterion was monitored to avoid over-fitting. Analyses were conducted using SPSS 17, AMOS (for SEM) and Stata 9IC (to adjust for data clustering in the regression models).

## Results

### Descriptive characteristics

Table 1 shows descriptive statistics. Unlike farmers, about 60% of the general community sample lived in a large metropolitan city. Australians in the general community sample were more likely than farmers to have a university education and farmers were more likely to be male, married and in full-time work. A substantial proportion of the general community sample were not in the workforce (unlike farmers, who were selected by occupation) probably because most were aged 50+ and likely retired. Consequently, the general community sample somewhat over-represents older Australians. Farmers also tended to be mature-aged, which is consistent with the profile of Australian farmers; in 2006, the median age of farmers was 52 and more than two-fifths were over 55 [34]. Although household income was similarly distributed across both samples, farmers clustered more tightly within a low-middle income bracket of $30,000–$79,999.

**Table 1:** Descriptive statistics

**Item**		**Farmers**	**General community**
**Socio-economic**			
Age (%)	18-29 yrs	8.20	12.55
	30-49 yrs	47.80	38.41
	50+ yrs	44.00	49.05
Sex (%)	Male	72.80	49.96
	Female	27.20	50.04
Location (%)	Major metropolitan	0	58.35
	Remainder of State	100.00	41.65
Relationship status (%)	Married	75.80	56.19
	Single	24.20	43.81
Work status (%)	Full-time	80.60	42.23
	Part-time	19.40	19.12
	Not in paid work	-	38.65
Level of education (%)	Degree	14.20	31.75
	Diploma	38.80	33.83
	Year 11	20.60	16.38
	Year 10	17.20	11.31
	Year 9 or below	9.20	6.73
Household income (%)	< $30,000	17.40	21.03
	$30,000–$79,999	50.20	42.81
	$80,000+	32.40	36.16
**Satisfaction**			
Overall (M, SD)		74.55^b^ (17.83)	**77.25** (16.64)
Domain (M, SD)	Community connectedness	73.87^a^ (18.64)	69.57 (19.92)
	Relationships	**83.17**^**a**^ (17.63)	79.49 (22.79)
	Safety	83.61^a^ (16.54)	78.94 (17.84)
	Standard of living	74.46^b^ (16.91)	**77.66**^**a**^ (16.65)
	Future security	66.89^b^ (21.34)	71.39 (20.12)
	Health	76.80^a^ (17.67)	74.33 (19.22)
	Achieving in life	72.36 (18.34)	**74.09**^**a**^ (18.54)
	Religion/spirituality	65.34^b^ (26.24)	68.23^b^ (26.21)

Note. Bolded means for life satisfaction indicate the mean is significantly higher than in the comparison sample in the next column, when controlling for demographic characteristics, p < .05.

^a^Mean value is higher than Australian norm, p < .05.

^b^Mean value is lower than Australian norm, p < .05.

The profile of satisfaction in the general community sample was very similar to 2008 Australian norms [35], however the current community sample was more satisfied with standard of living and achieving in life and was less satisfied with religion/spirituality. The farmer sample differed from Australian norms in a way that was also consistent with the pattern of mean differences between farmers and the general community sample. While adjusted comparisons indicated that many of the mean differences were due to demographic characteristics, it was notable that farmers’ overall satisfaction was significantly lower than the general community sample even when adjusting for confounders (for more detail contact corresponding author).

### Combining satisfaction domains into psychological life satisfaction

Unadjusted correlations established that, for farmers and general community Australians, there were significant associations between all combinations of the eight domains, although some relationships were stronger than others (see Additional file 1, Appendix B, Tables B1—B2). However, for both samples, when psychological satisfaction was regressed on the eight domains, several domains failed to explain a significant amount of unique variance (Table 2). There was also a sizeable discrepancy (.15—.41) between the size of domain-overall correlations and domain-overall semi-partial correlations, indicating a high level of covariance between different domain-overall satisfaction relationships. These results gave preliminary support for hypothesis 1; that different domains would cohere into overall psychological life satisfaction, with some domains being more influential.

**Table 2:** Regression predicting psychological satisfaction by domain satisfaction for farmers and the general community sample

	**Farmers (R**^**2 =**^**.47)**						
	***B***	***SE B***	**95% CI B**		***β***	***r***	***sr***
			**Lower**	**Upper**			
Relationships	.13	.03	.06	.19	.13***	.38	.11
Community connectedness	-.002	.04	-.09	.08	-.002	.29	-.002
Religion/ spirituality	.01	.03	-.05	.07	.02	.17	.02
Health	.05	.03	-.01	.11	.05	.35	.05
Safety	.08	.06	-.04	.19	.07	.37	.06
Future security	.09	.04	.02	.17	.11*	.48	.09
Standard of living	.09	.07	-.06	.23	.08	.45	.06
Achieving in life	.43	.06	.30	.55	.44***	.64	.33
	**General community (R2=.54)**						
	***B***	***SE B***	**95% CI B**		***β***	***r***	***sr***
			**Lower**	**Upper**			
Relationships	.15	.02	.11	.18	.20***	.49	.17
Community connectedness	.05	.03	-.01	.10	.06	.38	.05
Religion/ spirituality	-.002	.02	-.04	.04	-.002	.22	-.002
Health	.12	.03	.06	.18	.14***	.46	.12
Safety	-.01	.02	-.06	.04	-.01	.31	-.01
Future security	.09	.03	.02	.16	.11**	.54	.08
Standard of living	.19	.02	.15	.24	.19***	.55	.15
Achieving in life	.28	.03	.21	.35	.31***	.64	.23

*p ≤.05, **p ≤.01, ***p ≤.001.

Note. These regressions are unadjusted for demographic characteristics; their purpose is to examine covariance between life satisfaction domains. The semi-partial coefficients (sr) report the unique variance that each predictor shares with overall life satisfaction, adjusting for covariance between different predictors.

**Figure 1:**
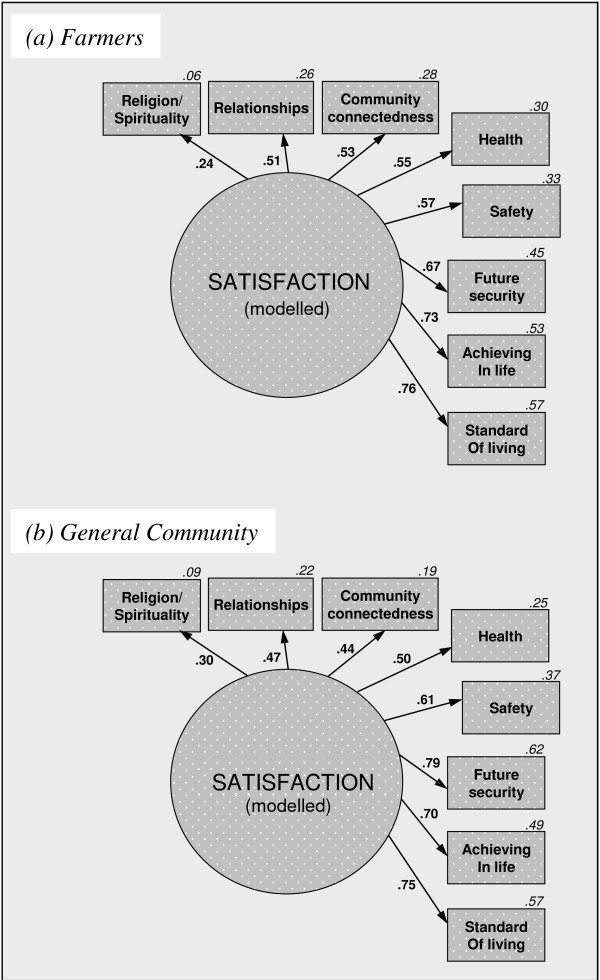
**Single satisfaction construct modelled from domains for the farmer and general community samples.** Note 1. For ease of reading, error terms and pathways <.20 have been removed; they are included in Figure C1 (See Additional file 1, Appendix C). Note 2. Supplementary analyses confirmed that the unitary satisfaction constructs were strongly correlated with the explicit psychological satisfaction measure. Further details are available from the corresponding author.

To quantify how co-varying domains might cohere into a unitary construct of overall psychological satisfaction, we built one-factor congeneric models (Figure 1). Within both samples, standard of living, achieving in life and future security loaded strongly onto a unitary construct. Supporting hypothesis 1, adequate fit statistics were obtained for the general community sample (*χ*^2^=49.19,p<.001,CFI=.90,RMSEA=.04), and for the farmer sample (*χ*^2^=40.84,p=.004,CFI=.81,RMSEA=.05).

### Supra-domains of satisfaction

**Figure 2:**
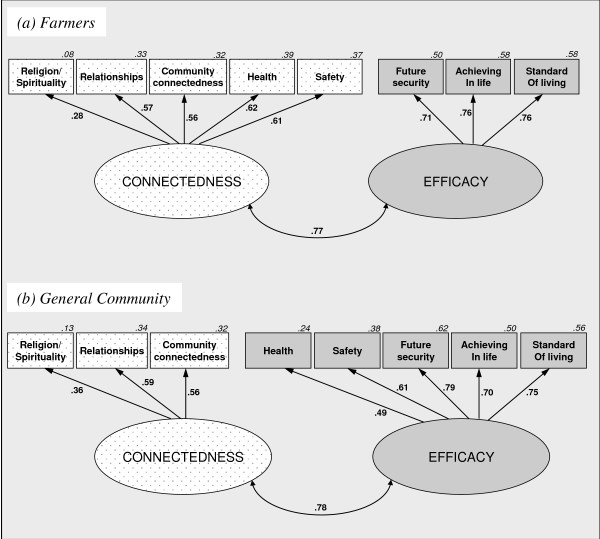
**Supra-domains of satisfaction modelled from domains for the farmer and general community samples.** Note 1. For clarity error terms and pathways <.20 have been removed, for full models see Figure B2 (See Additional file 1, Appendix C). Note 2. Separate analyses confirmed that the supra-domain satisfaction factors were strongly correlated with the explicit psychological satisfaction measure.

We then used exploratory factor analysis to investigate the presence of supra-domains (see Additional file 1, Appendix B, Table B3). The farmer sample returned a two-factor solution consistent with the hypothesised supra-domains of connectedness and effectiveness. The general community sample initially returned a single factor solution consistent with Figure 1b. However, when a 2-factor solution was requested, the returned factors closely resembled those found within the farmer sample. One-factor congeneric models were next used to confirm supra-domain structure; supra-domains were then correlated to produce the models shown in Figure 2.

For farmers, the connectedness supra-domain comprised satisfaction with relationships, community connectedness, religion/spirituality, safety and health. It was strongly positively correlated with the efficacy supra-domain, which comprised satisfaction with future security, standard of living and achieving in life. Strong model fit statistics were obtained, *χ*^2^=19.14,p=.45,CFI=1.00,RMSEA=.004.

Supra-domain composition for general community Australians was very similar to that for farmers, except that satisfaction with health and with safety loaded onto efficacy, rather than onto connectedness. Again, the supra-domains were strongly positively correlated and the model fit the data well, *χ*^2^=41.77,p<.001, CFI=.92,RMSEA=.04. Notably, these general community fit indices were slightly better for the two-factor model than the unitary model, and the farmer fit indices were markedly better.

### Supra-domains predicting psychological life satisfaction

To examine relationships between connectedness, efficacy and psychological life satisfaction, using SEM, we concurrently modelled reverse pathways between the supra-domains, and between each supra-domain and the measured overall satisfaction item (i.e. directional arrows were initially tested in both directions). Socioeconomic variables were included to control for their likely relationship with the supra-domains.

**Figure 3:**
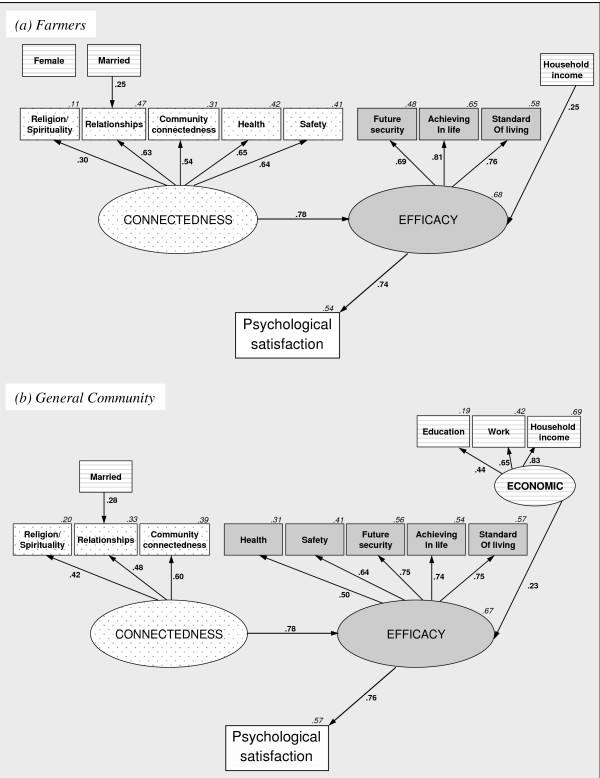
**Supra-domains predicting psychological satisfaction for the farmer and general community samples.** Note 1. For clarity error terms and relationships <.20 have been removed, for full models see Figure B3 (See Additional file 1, Appendix C). Note 2. Separate analyses confirmed that the supra-domain satisfaction factors were strongly correlated with the explicit psychological satisfaction measure.

The final models for farmers and general community Australians produced identical underlying relationships (Figure 3). For both groups, connectedness predicted efficacy, which predicted overall satisfaction. Other pathways between these three became non-significant when modelled concurrently, despite connectedness having a strong bivariate association with the overall satisfaction item. To confirm the plausibility of the mediation pathway, we used factor weights derived from the SEM to construct weighted composite scores for each supra-domain which we regressed on overall satisfaction to test for the mediation pathways shown in Figure 3. Although the composite scores produced partial (~50%) rather than full mediation, the replicated effects were similarly strong and significant (see Additional file 1, Appendix B, Table B4). It is worth noting that the regressions also showed no evidence of multi-colinearity, supporting the conceptually meaningful nature of the mediation.

For both samples, the final SEM models were less parsimonious than those presented in Figure 2, which resulted in poorer, though still acceptable, model fit (farmers: *χ*^2^=195.06,p<.001,CFI=.88,RMSEA=.05, general community: *χ*^2^=79.37, p<.01,CFI=.87,RMSEA=.03). Consistent with other population health research, in the general community sample, education, paid employment and household income clustered together to form a single construct [36,37], which positively predicted efficacy supra-domain satisfaction. In contrast, farmers’ efficacy satisfaction was simply predicted by household income. For both groups, being married predicted greater satisfaction with relationships.

## Discussion

The aim of this study was to identify key concepts and relationships that can be used to understand, predict and affect psychological life satisfaction, for which we tested three hypotheses. The support these hypotheses received gave novel insight into life satisfaction, and in particular provided a way of quantifying differences and similarities between populations in terms of the way psychological life satisfaction is structured. The benefits of these findings for designing health promotion programs and preventative strategies are discussed.

Our test of Hypothesis 1 was, essentially, a test of the current academic view that discrete domains will cohere into overall psychological life satisfaction, although different domains may be more influential than others. This hypothesis was broadly supported by findings in the general community sample. However, in the farmer sample, although the model of unitary sense of psychological satisfaction received acceptable fit statistics, the exploratory factor analysis indicated that the domains did not simply combine into a unitary sense of psychological satisfaction.

Hypothesis 2 tested the novel idea that perceived satisfaction with different life domains will covary into a small number of supra-domains. In support of hypothesis 2, the same two supra-domains, encapsulating connectedness and efficacy, were evident in both the farmer and general community samples, showing good fit within both samples. The *consistency across* samples in the way that six of the eight domains loaded onto the same supra-domains supported the robustness of the supra-domain constructs, and highlighted that the needs and priorities of drought-affected farmers are similar to those of all Australians. However, the *differences between* supra-domains for the two samples were also instructive: farmers saw health and safety as a part of connectedness, while general community Australians saw these domains as part of efficacy.

Farmers in Australia typically work on large remote properties and rely heavily on their farming neighbours (i.e., on their connectedness) for health and safety information, training and emergencies [38]. Industry “field-days”, trade shows and events are key times for farmers to meet and learn about the safe operation of new equipment. These community events are also a time when farmers engage with health providers, who frequently use field days and trade shows for opportunistic health interventions. For many farmers these field days are the only time that they interact with others besides the few people on their farm and a few suppliers in the township closest to their remote landholding. In contrast, most other Australians are more able to rely on systematised infrastructures providing access to services such as doctors, police, fire and ambulance services. The differences between the two models were congruent with contextual differences between the lives of Australian farmers, where health and safety are directly related to connectedness, versus the lives of most Australians, where they are not.

Being able to model the impact of contextual differences on the way psychological life satisfaction is structured is useful for understanding the nature of psychological life satisfaction in different populations. However, Hypothesis 3 went one step further to investigate whether there are also unique relationships between domain-specific perceptions of satisfaction and psychological life satisfaction. Understanding these patterns could give important general insights on how to promote (and hence better) health behaviours and mental health.

Hypothesis 3’s prediction of unique relationships was supported. For farmers and general community Australians, using SEM to test reverse pathways showed that satisfaction with the connectedness supra-domain strongly predicted satisfaction with the efficacy supra-domain, while efficacy only weakly predicted connectedness. Further, when both were tested simultaneously, the pathway from efficacy to connectedness was not significant. Even more striking, efficacy directly predicted overall psychological satisfaction, while connectedness did not. Although the influence of connectedness on psychological satisfaction was strongly consistent with social capital theory: [28,39], the pathway was entirely indirect, mediated by satisfaction with efficacy.

The result indicates that efficacy domains play a central and direct role in a person’s wellbeing, which is consistent with Bandura’s theory of self-efficacy [40]. The direction of the connectedness-to-efficacy path may be because connectedness provides resources and opportunities that can be used to achieve goals, improve standard of living and secure futures [39], but obtaining these outcomes does not necessarily lead to improved connectedness.

However, despite the proximal importance of people’s sense of efficacy, health policy interventions may struggle to affect it (i.e. improve achievement satisfaction and future security etc.). Instead, our findings suggest an alternative, and perhaps more achievable, strategy by showing that both efficacy and psychological wellbeing are strongly associated with satisfaction with connectedness. Although the study is a cross-sectional one, the pattern of associations we found suggests that connectedness-building interventions may be able to increase efficacy and *indirectly* drive psychological satisfaction and the multiple wellbeing outcomes that satisfaction reliably predicts.

Further, modelling the composition of connectedness and efficacy supra-domains can potentially inform the tailoring of connectedness-building interventions for specific populations. For example, our results suggest that initiatives explicitly focussed on safety and health may be a good way to promote connectedness amongst Australian farmers because satisfaction in these domains is associated with satisfaction with relationships and community connectedness.

### Limitations

Although the relationships reported here are consistent with existing theory, they may be limited to the Australian samples examined; as the results showed, contextual effects do occur. Consequently, establishing the findings’ generaliseability to other countries would require similar research utilising a range of representative samples. It would also be beneficial to examine the modelled relationships longitudinally and test the models’ predictive power across time. These issues are unavoidable limitations of the current work but are also important directions for future cross-contextual research on the structure of life satisfaction and wellbeing.

## Conclusions

Our findings indicate that while it is reasonable to examine life satisfaction as a unitary concept, there is also scope to examine supra-domains as a way of better understanding the architecture of life satisfaction. Our study of these supra-domains most clearly provides a novel insight about how to help protect the life satisfaction and wellbeing of people living in Australia. However, the scope of our contribution is potentially greater because the theory and methods applied in our research provide a way of analysing life satisfaction that can be tested and applied within any population, with benefits for health policy.

## Competing interests

The authors declare that they have no competing interests.

## Authors’ contributions

LVO designed and performed the analyses, and drafted the manuscript. HLB substantially contributed to the design and conduct of the analyses, and critically revised the manuscript for important intellectual content. AH substantially contributed in the design and coordination of the study and revised the manuscript for important intellectual content. All authors read and approved the final manuscript.

## Pre-publication history

The pre-publication history for this paper can be accessed here:

http://www.biomedcentral.com/1471-2458/12/976/prepub

## Supplementary Material

Additional file 1 **Appendices A, B & C.** Supplementary material regarding (a) sampling procedure and missing data, (b) associations between measures, and (c) relationships in the SEM models that were <.2 in strength. Click here for file
