# Supplementary material for: The structure of psychological life satisfaction: insights from farmers and a general community sample in Australia
Source: BMC Public Health. 2012 Nov 14;12:976. doi: 10.1186/1471-2458-12-976 (PMC3527324; doi:10.1186/1471-2458-12-976)
Supplement: Additional file 1 — Appendices A, B & C. Supplementary material regarding (a) sampling procedure and missing data, (b) associations between measures, and (c) relationships in the SEM models that were <.2 in strength. [file 1471-2458-12-976-S1.doc]

**Appendix A – SAMPLING PROCEDURE AND MISSING DATA**

The farmer population in drought in 2008 was 308,000, and the general population of Australia was 21,180,600. Sample sizes were based on power analyses and past research examining the populations considered in the current study. To conduct the data collection, trained interviewers from the polling firm Newspoll were briefed by the Australian Bureau of Rural Sciences and then made telephone calls to households randomly selected from the sampling regions’ electoral rolls (see tables A1 and A2). The general community sample was collected between 18 and 20 July 2008, and the farmer sample was collected between 25 and 27 July 2008. Non-response occurred where eligible adults declined to participate in a telephone interview, and/or where they were unavailable after a second call-back.

When participants in the study declined to answer a question in interview, their response was treated as a missing data point in the analyses (see table A3). After missing data were imputed and core analyses conducted, two additional sensitivity analyses were performed to assess the validity of the imputed data. Firstly, because there was substantial missing data for satisfaction with religion/spirituality in both samples, the t-test comparing satisfaction across samples was performed using original data only; this replicated the non-significant result found using the imputed data, *t*(1,174)=.99, *p*=.32. Secondly, the regression analysis conducted to test the mediation path between connectedness, efficacy and psychological life satisfaction, which included household income as a covariate, was also performed using original data only. The results are presented in table A4; they also replicate those reported using imputed data.

Table A1. Sampling regions for the farmer sample.

| State | Region | Frequency | | % of total |
| --- | --- | --- | --- | --- |
| Queensland | Longreach | 8 | 1.60 | |
|  | Emerald | 23 | 4.60 | |
|  | Gatton | 38 | 7.60 | |
|  | Murweh | 14 | 2.80 | |
| New South Wales | Bourke | 10 | 2.00 | |
|  | Inverell | 23 | 4.60 | |
|  | Gilgandra | 23 | 4.60 | |
|  | Forbes | 30 | 6.00 | |
|  | Griffith | 72 | 14.40 | |
| Victoria | Mildura | 36 | 7.20 | |
|  | Greater Shepparton East | 20 | 4.00 | |
|  | Greater Shepparton West | 38 | 7.60 | |
|  | East Gippsland/Bairnsdale | 11 | 2.20 | |
|  | Buloke-North | 21 | 4.20 | |
|  | Central highlands | 11 | 2.20 | |
| Western Australia | Wongan-Ballidu | 9 | 1.80 | |
|  | Morawa | 4 | .80 | |
|  | Esperance | 27 | 5.40 | |
|  | Merredin | 9 | 1.80 | |
| South Australia | Le Hunte | 11 | 2.20 | |
|  | Renmark Paringa | 9 | 1.80 | |
|  | Tatiara | 46 | 9.20 | |
|  | Orroroo/Carrieton | 7 | 1.40 | |
| Total |  | 500 | 100.00 | |

Table A2. Sampling regions for the general community sample.

| State | Region | Frequency | % of total |
| --- | --- | --- | --- |
| Queensland | Brisbane | 100 | 8.31 |
|  | Remainder of state | 101 | 8.40 |
| New South Wales | Sydney | 200 | 16.63 |
|  | Remainder of state | 132 | 10.97 |
| Australian Capital Territory |  | 18 | 1.50 |
| Victoria | Melbourne | 200 | 16.63 |
|  | Remainder of state | 100 | 8.31 |
| South Australia | Adelaide | 100 | 8.31 |
|  | Remainder of state | 50 | 4.16 |
| Western Australia | Perth | 102 | 8.48 |
|  | Remainder of state | 50 | 4.16 |
| Tasmania |  | 50 | 4.16 |
| Total |  | 1203 | 100.00 |

Table A3. Missing data in the farmer and general community samples.

| Item | Farmers | | General community | | |
| --- | --- | --- | --- | --- | --- |
| Socio-economic | Frequency | % of total | Frequency | | % of total |
| Age | 0 | - | 0 | | - |
| Sex | 0 | - | 0 | | - |
| Relationship status | 0 | - | 0 | | - |
| Work status | 2 | 0. 04 | 3 | | 0.03 |
| Level of education | 4 | 0.08 | 31 | | 2.58 |
| Household income | 64 | 12.80 | 220 | | 18.29 |
| Satisfaction |  |  |  | |  |
| Psychological (overall) | 1 | 0.02 | 8 | .07 | |
| Community connectedness | 1 | 0.02 | 21 | 1.75 | |
| Relationships | 6 | 1.20 | 16 | 1.33 | |
| Safety | 6 | 1.20 | 7 | 0.06 | |
| Standard of living | 1 | 0.02 | 4 | 0.03 | |
| Future security | 6 | 1.20 | 24 | 0.02 | |
| Health | 1 | 0.02 | 2 | 0.02 | |
| Achieving in life | 3 | 0.06 | 18 | 0.01 | |
| Religion/spirituality | 80 | 16.00 | 179 | 14.88 | |

Table A4. Sensitivity regression using original data (missing values not imputed) to predict psychological satisfaction using socio-economic variables and the factor-weighted supra-domain scales for farmer and general community samples.

|  |  | Farmers | | | | | General community | | | | |
| --- | --- | --- | --- | --- | --- | --- | --- | --- | --- | --- | --- |
|  | Items | B | SE B | 95% CI B | | β | B | SE B | 95% CI B | | β |
|  |  |  |  | Lower | Upper |  |  |  | Lower | Upper |  |
| Step 1 |  |  |  |  |  |  |  |  |  |  |  |
|  | Age | - | - | - | - | - | .53 | .13 | .22 | .84 | .09** |
|  | Household income (eq.) | 1.03 | .42 | .17 | 1.91 | .12* | 1.14 | .21 | .68 | 1.60 | .13*** |
|  | Connected | .31 | .02 | .27 | .35 | .62*** | .32 | .02 | .27 | .37 | .45*** |
| Step 2 |  |  |  |  |  |  |  |  |  |  |  |
|  | Age | - | - | - | - | - | .47 | .10 | .25 | .69 | .05* |
|  | Household income (eq.) | -.05 | .41 | -.91 | .81 | -.01 | .45 | .16 | .09 | .82 | .08*** |
|  | Connected | .15 | .03 | .09 | .21 | .30*** | .13 | .02 | .08 | .18 | .18*** |
|  | Efficacy | .27 | .04 | .19 | .34 | .54*** | .20 | .01 | .17 | .22 | .53*** |
| R^2^ |  | .48 | | | | | .51 | | | | |

Note. Efficacy predicted Connected in both the farmer sample, B = .52 [.40 .63], SE B = .05, β = .52, p < .001, and the general community sample, B = .30 [.25 .34], SE B = .02, β = .57, p < .001.

Note 2. All socioeconomic variables were included in the regressions, non-significant results have been deleted.

**p <.01, ***p <.001

**Appendix B – Supplementary Tables**

Table B1. Correlations between PWI items for farmers.

| Domain | SL | Hth. | Ach. | Rel. | Saf. | CC | FS |
| --- | --- | --- | --- | --- | --- | --- | --- |
| Standard of living (SL) | 1.00 |  |  |  |  |  |  |
| Health (Hth.) | 0.30*** | 1.00 |  |  |  |  |  |
| Achieving in life (Ach.) | 0.52*** | 0.38*** | 1.00 |  |  |  |  |
| Relationships (Rel.) | 0.29*** | 0.39*** | 0.35*** | 1.00 |  |  |  |
| Safety (Saf.) | 0.39*** | 0.34*** | 0.37*** | 0.31*** | 1.00 |  |  |
| Community connectedness (CC) | 0.35*** | 0.34*** | 0.33*** | 0.35*** | 0.32*** | 1.00 |  |
| Future security (FS) | 0.54*** | 0.29*** | 0.56*** | 0.26*** | 0.37*** | 0.26*** | 1.00 |
| Religion/spirituality(RS) | 0.10* | 0.16*** | 0.19*** | 0.20*** | 0.17*** | 0.19*** | 0.14** |

***p <.001

Table B2. Correlations between PWI items for general community sample.

| Domain | SL | Hth. | Ach. | Rel. | Saf. | CC | FS |
| --- | --- | --- | --- | --- | --- | --- | --- |
| Standard of living (SL) | 1.00 |  |  |  |  |  |  |
| Health (Hth.) | 0.36*** | 1.00*** |  |  |  |  |  |
| Achieving in life (Ach.) | 0.53*** | 0.45*** | 1.00 |  |  |  |  |
| Relationships (Rel.) | 0.33*** | 0.28*** | 0.40*** | 1.00 |  |  |  |
| Safety (Saf.) | 0.42*** | 0.30*** | 0.32*** | 0.17*** | 1.00 |  |  |
| Community connectedness (CC) | 0.28*** | 0.27*** | 0.40*** | 0.36*** | 0.28*** | 1.00 |  |
| Future security (FS) | 0.60*** | 0.38*** | 0.55*** | 0.37*** | 0.44*** | 0.34*** | 1.00 |
| Religion/spirituality(RS) | 0.18*** | 0.21*** | 0.24*** | 0.21*** | 0.19*** | 0.32*** | 0.21*** |

***p <.001

Table B3. Rotated factor loadings from exploratory factor analysis of domains of satisfaction for farmers and general community sample.

| Satisfaction with life domain | Farmers | | |  | General community | |
| --- | --- | --- | --- | --- | --- | --- |
|  | 1 | 2 |  | | 1 | 2 |
| Relationships | .65 |  |  | |  | .37 |
| Community connectedness | .53 |  |  | |  | .69 |
| Religion spirituality | .32 |  |  | |  | .46 |
| Health life | .59 |  |  | | .39 |  |
| Safety | .35 |  |  | | .51 |  |
| Future security |  | -.86 |  | | .79 |  |
| Standard of living |  | -.64 |  | | .84 |  |
| Achieving in life |  | -.61 |  | | .55 |  |

Note 1. Maximum Likelihood factoring with oblimin Kaiser Normalization rotation.

Note 2. Farmers goodness of fit: χ²(13) = 11.12, p= .60, Bartlett’s test = χ²(28) = 923.18, p <.001, KMO = .85; general community sample: χ²(13) = 63.77, p <.001, Bartlett’s test = χ²(28) = 2490.07, p <.001, KMO = .86.

Table B4. Variance explained from exploratory factor analysis of domains of satisfaction for the farmer sample.

| Factor | Initial Eigenvalues | | | Extraction Sums of Squared Loadings | | | Rotation Sums of Sqd. Loadings^a^ |
| --- | --- | --- | --- | --- | --- | --- | --- |
|  | Total | % of Variance | Cumulative % | Total | % of Variance | Cumulative % | Total |
| 1 | 3.27 | 40.90 | 40.90 | 2.70 | 33.74 | 33.74 | 2.23 |
| 2 | 1.04 | 12.96 | 53.86 | 0.47 | 5.91 | 39.65 | 2.39 |
| 3 | 0.85 | 10.68 | 64.54 |  |  |  |  |
| 4 | 0.69 | 8.56 | 73.10 |  |  |  |  |
| 5 | 0.67 | 8.33 | 81.43 |  |  |  |  |
| 6 | 0.60 | 7.49 | 88.93 |  |  |  |  |
| 7 | 0.47 | 5.83 | 94.75 |  |  |  |  |
| 8 | 0.42 | 5.25 | 100.00 |  |  |  |  |
| Extraction Method: Maximum Likelihood, extracted factors for eigenvalues > 1. | | | | | | | |
| ^a^When factors are correlated, sums of squared loadings cannot be added to obtain a total variance. | | | | | | | |

Table B5. Total variance explained from exploratory factor analysis of domains of satisfaction for the general community sample.

| Factor | Initial Eigenvalues | | | Extraction Sums of Squared Loadings | | | Rotation Sums of Sqd. Loadings^a^ |
| --- | --- | --- | --- | --- | --- | --- | --- |
|  | Total | % of Variance | Cumulative % | Total | % of Variance | Cumulative % | Total |
| 1 | 3.42 | 42.72 | 42.72 | 2.87 | 35.90 | 35.90 | 2.72 |
| 2 | 1.00 | 12.49 | 55.21 | .41 | 5.10 | 41.00 | 2.02 |
| 3 | .84 | 10.45 | 65.66 |  |  |  |  |
| 4 | .72 | 8.95 | 74.60 |  |  |  |  |
| 5 | .65 | 8.06 | 82.66 |  |  |  |  |
| 6 | .57 | 7.12 | 89.78 |  |  |  |  |
| 7 | .42 | 5.25 | 95.03 |  |  |  |  |
| 8 | .40 | 4.97 | 100.00 |  |  |  |  |
| Extraction Method: Maximum Likelihood, two factors requested. | | | | | | | |
| ^a^When factors are correlated, sums of squared loadings cannot be added to obtain a total variance. | | | | | | | |

Table B6. Regressions to replicate the final SEM models, predicting psychological satisfaction using socio-economic variables and the factor-weighted supra-domain scales for farmer and general community samples.

|  |  | Farmers | | | | | General community | | | | |
| --- | --- | --- | --- | --- | --- | --- | --- | --- | --- | --- | --- |
|  | Items | B | SE B | 95% CI B | | β | B | SE B | 95% CI B | | β |
|  |  |  |  | Lower | Upper |  |  |  | Lower | Upper |  |
| Step 1 |  |  |  |  |  |  |  |  |  |  |  |
|  | Age | - | - | - | - | - | .49 | .16 | .14 | .83 | .08** |
|  | Household income (eq.) | 1.23 | .38 | .46 | 2.02 | .14** | 1.37 | .14 | .14 | .83 | .15*** |
|  | Connected | .27 | .02 | .23 | .31 | .50*** | .33 | .02 | .28 | .38 | .48*** |
| Step 2 |  |  |  |  |  |  |  |  |  |  |  |
|  | Age | - | - | - | - | - | .48 | .11 | .23 | .73 | .08*** |
|  | Household income (eq.) | -.03 | .39 | -.78 | .72 | -.003 | .55 | .15 | .21 | .89 | .06** |
|  | Connected | .11 | .02 | .07 | .16 | .21*** | .14 | .02 | .10 | .18 | .12*** |
|  | Efficacy | .26 | .03 | .20 | .33 | .54*** | .20 | .01 | .17 | .23 | .54*** |
| R^2^ |  | .43 | | | | | .50 | | | | |

Note 1. Efficacy predicted Connected in both the farmer sample, B = .50 [.40 .61], SE B = .05, β = .56, p < .001, and the general community sample, B = .28 [.25 .31], SE B = .01, β = .54, p < .001.

Note 2. All socioeconomic variables were included in the regressions, non-significant results have been deleted.

**p <.01, ***p <.001

**Appendix C – Supplementary Figures**

Figure C1. Satisfaction as a unitary construct modelled as a single one-factor congeneric model using domain scores for the farmer and general community samples and showing correlated error terms.

SATISFACTION


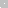


SATISFACTION


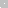


Note. Separate analyses confirmed that the psychological satisfaction factors were strongly correlated with the explicit psychological satisfaction measure.

Figure C2. Supra-domains of satisfaction modelled as correlated one-factor congeneric models using domain scores for the farmer and general community samples and showing correlated error terms.

Note. Separate analyses confirmed that the supra-domain satisfaction factors were strongly correlated with the explicit psychological satisfaction measure.

Figure C3. Modelled supra-domains of satisfaction predicting psychological satisfaction for the farmer and general community samples and showing correlated error terms

Note. Separate analyses confirmed that the supra-domain satisfaction factors were strongly correlated with the explicit psychological satisfaction measure.

Psychological

satisfaction

Psychological

satisfaction
